# EN1 Regulates Cell Growth and Proliferation in Human Glioma Cells via Hedgehog Signaling

**DOI:** 10.3390/ijms23031123

**Published:** 2022-01-20

**Authors:** Jinchun Chang, Chenjia Guo, Jianyu Li, Zhangqian Liang, Yankai Wang, Anliang Yu, Runze Liu, Yuting Guo, Jian Chen, Song Huang

**Affiliations:** 1 College of Life Sciences, Beijing Normal University, Beijing 100875, China; changjinchun@nibs.ac.cn; 2 National Institute of Biological Sciences, Beijing 102206, China; wangyankai@nibs.ac.cn (Y.W.); yuanliang@nibs.ac.cn (A.Y.); liurunze@nibs.ac.cn (R.L.); guoyuting@nibs.ac.cn (Y.G.); 3 Chinese Institute for Brain Research, Beijing 102206, China; guochenjia@cibr.ac.cn (C.G.); lijianyu@cibr.ac.cn (J.L.); liangzhangqian@cibr.ac.cn (Z.L.); 4 Institute of Functional Nano and Soft Materials (FUNSOM) & Collaborative Innovation Center of Suzhou Nano Science and Technology, Soochow University, Suzhou 215123, China

**Keywords:** glioblastoma, Engrailed, irradiation sensitivity, ROS, Hedgehog

## Abstract

Glioblastoma is an aggressive cancer of the nervous system that accounts for the majority of brain cancer-related deaths. Through cross-species transcriptome studies, we found that Engrailed 1 (EN1) is highly expressed in serum-free cultured glioma cells as well as glioma tissues, and increased expression level predicts a worse prognosis. EN1 controls glioma cell proliferation, colony formation, migration, and tumorigenic capacity in vivo. It also influences sensitivity of glioma cells to γ-ray irradiation by regulating intracellular ROS levels. Mechanistically, EN1 influences Hedgehog signaling by regulating the level of Gli1 as well as primary cilia length and the primary cilia transport-related protein TULP3. In conclusion, we demonstrate that EN1 acts as an oncogenic regulator that contributes to glioblastoma pathogenesis and could serve as a diagnostic/prognostic marker and therapeutic target for glioblastoma.

## 1. Introduction

Gliomas are the most common types of brain cancer, and glioblastoma is the most aggressive form of cancer that begins within the brain [1]. It accounts for more than 60% of all brain tumors in adults, and has been designated as a Grade IV tumor of the central nervous system by the World Health Organization [2]. The median survival of glioblastoma patients is generally 14–16 months, with fewer than 3–5% of patients surviving >5 years [3]. Currently, the standard treatment for glioblastoma is maximal safe resection followed by radiotherapy and chemotherapy with temozolomide [4]. In spite of intense efforts to optimize the treatment of gliomas over the past decades, no significant advancement in patient survival has been achieved. Notably, glioblastoma is also prone to chemotherapy and radiation resistance [5].

The gene Engrailed was first discovered by Ecker in a spontaneous mutant of *Drosophila melanogaster* in 1929, and it has been identified as a conserved gene cassette throughout holometabolous insects [6]. Vertebrates have two Engrailed genes (EN1 and EN2), and these homeobox-containing transcription factors function in regulating early embryonic development [7]. During the development of mouse, EN1 and EN2 have overlapping functions in regulating formation of additional fissures and for extensive cerebellar growth [8,9], but mutations in EN1 and EN2 produce different phenotypes [10]. In humans, aberrant expression of these genes has been associated with the pathogenesis of many types of tumors, including breast cancer [11,12], prostate cancer [13,14], colorectal cancer [15], and ovarian cancer [16,17]. Elevated EN2 expression in particular has been associated with poor survival, and this gene has been proposed as a biomarker for breast cancer screening [18]. The molecular basis of Engrailed-mediated pathogenic impacts in glioma remains unknown.

The Hedgehog signaling pathway was first identified in the *Drosophila melanogaster*; it is an evolutionarily conserved pathway of signal transmission from the cell membrane to the nucleus [19,20]. Its plays a significant role in the normal embryonic development and organogenesis of almost all organs in mammals [21]. Aberrant activation of Hedgehog signaling is one of the key oncogenic events in pediatric brain tumor medulloblastomas [22] and is also involved in glioma pathogenesis and glioma stem-cell maintenance [23].

Here, seeking to better understand the aggressive nature of glioblastomas, we investigated glioma stem cells obtained from isolated stem-cell cultures of mouse spontaneous glioma tumor samples [24]. Comparative transcriptomics of mouse glioma and normal stem cells, glioblastoma tumors, and normal tissues revealed that the mouse ortholog of Engrailed is highly expressed in gliomas. Our follow-up analyses ultimately indicate that EN1 is tumorigenic for gliomas, regulating the proliferation and growth of cancerous cells by modulating Hedgehog signaling activity.

## 2. Results

### 2.1. EN1 Is Upregulated in Glioblastoma and Is Related to Poor Patient Prognosis

**Figure 1:**
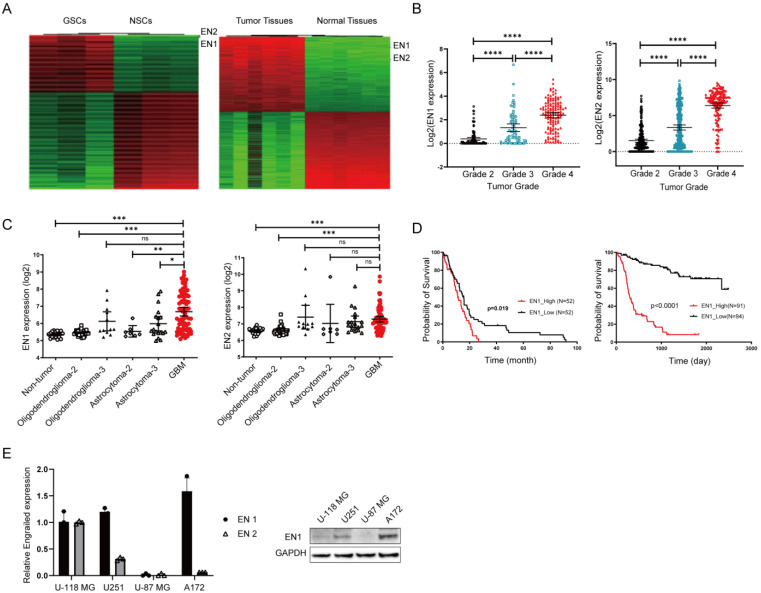
Elevated EN expression is associated with glioma cancer progression. (**A**) RNA-seq results for mouse glioma stem cells lines vs. neural stem cells (left) and for mouse brain tumor tissues vs. normal brain tissues (right). (**B**) The expression of EN1 (left) and EN2 (right) in glioma samples from TCGA database. Black, blue and red color is for Grade 2, 3 and 4 respectively. (**C**) Analysis using the Henry Ford Hospital dataset, showing correlation between the EN1 (left) and EN2 (right) gene expression levels and glioma patient tumor grade (○ for Non-tumor, □ and ▲ for Oligodendroglioma-2 and 3, ◊ and ∆ for Asreocytoma-2 and 3, red ● for GBM). (**D**) Correlation between glioma patient survival and the EN1 expression level from TCGA glioblastoma database (left) and the Chinese Glioma genome Atlas gliomas database (right). (**E**) For the indicated glioma cell lines, Engrailed 1 expression assessed by qPCR (left) and Engrailed 1 accumulation assessed by immunoblotting (right). * *p* < 0.05, ** *p* < 0.01, *** *p* < 0.001, **** *p* < 0.0001 and ns (no significant) according to Student’s *t*-test.

Glioma stem cells (GSCs) are believed to contribute to tumor maintenance, therapeutic resistance, and tumor recurrence [25,26]. In order to identify key molecules regulating GSC, we examined glioma stem cells obtained from mouse spontaneous glioma tumor samples [24]. Our transcriptomics analysis of glioma stem cells and normal neural stem cells (NSCs) indicated that Engrailed was the top-ranking differentially expressed gene between GSCs and NSCs (Figure 1A). We also found that the Engrailed gene was expressed at higher levels in mouse glioblastoma tumors than in normal tissues (Figure 1A), and it was mostly expressed by tumor cells (Figure S7).

To examine the potential clinical relevance of Engrailed expression in gliomas, we analyzed clinical sample data from TCGA database and found that Engrailed 1 expression was significantly increased in glioblastoma tissue samples as compared to normal tissue specimens and to other low-grade gliomas (*p* < 0.001, Figure 1B). Similar expression trends were detected in the datasets studied by Henry Ford Hospital [27] (Figure 1C), further indicating that increased EN1 expression is associated with higher glioma grade.

To further assess the correlation between expression level of EN1 and patient survival with glioblastoma, we performed Kaplan–Meier survival analysis on the basis of the EN1 expression levels of glioblastoma patients from TCGA databases and the Chinese genome project database [28]. The overall survival of glioblastoma patients with high levels of EN1 expression was significantly poorer than that of patients with lower EN1 expression (*p* < 0.01) (Figure 1D and Figure S1). These findings indicated that EN1 may be associated with progression of glioblastoma.

### 2.2. EN1 Knockdown Restrains the Proliferation and Migration of Glioblastoma Cells In Vitro and In Vivo

**Figure 2:**
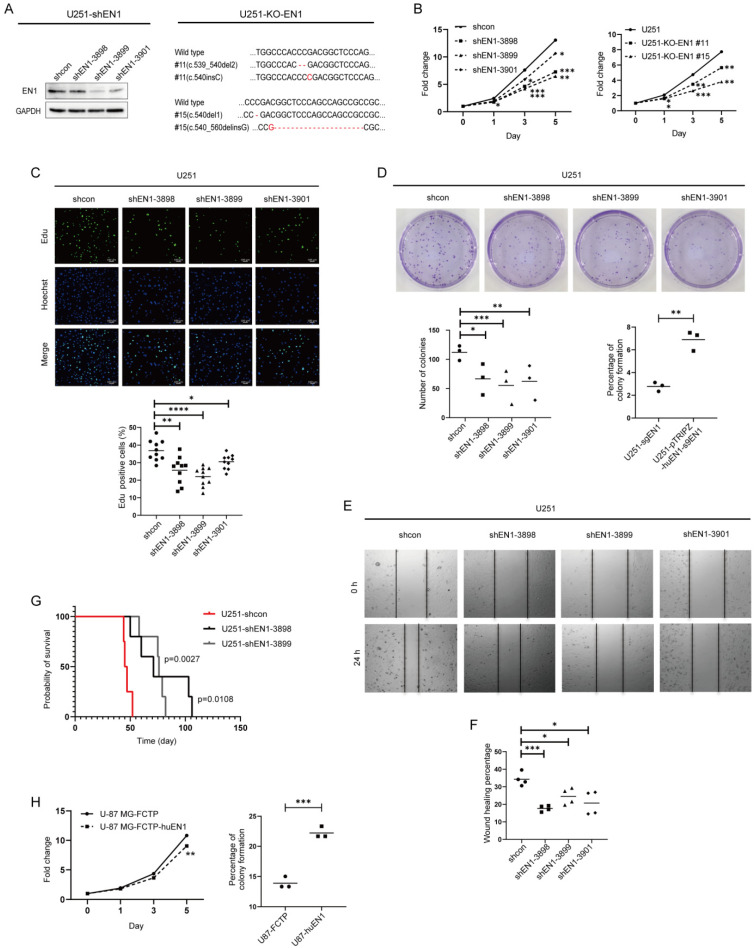
EN1 knockdown restrains the proliferation and migration of glioma cells. (**A**) Assessment of knockdown efficiency for EN1 with immunoblotting, and confirmation of EN1 knockout in U251 cells with immunoblotting and genome sequencing. Cell proliferation was assessed using CellTiter-Glo assays (**B**), 5-ethynyl-2′-deoxyuridine (Edu) assays (**C**), and colony formation assays (**D**) (● for shcon, ■ for shEN1-3898, ▲ for shEN1-3899, ◆ for shEN1-3901). (**E**) Cell migration was assessed with a wound-healing assay. (**F**) Quantification of wound-healing assay. (**G**) Survival curves for mice bearing xenograft tumors derived from shRNA-EN1 cells or scramble control cells. (**H**) Cell proliferation was assessed by CellTiter-Glo and colony formation assays in U-87 MG cells after EN1 overexpression. Scale bar: 100 μm. Data are presented as the mean ± SEM from three independent experiments. * *p* < 0.05, ** *p* < 0.01, *** *p* < 0.001, **** *p* < 0.0001 according to Student’s *t*-test.

To evaluate the functions of EN1 in glioma cells, we first assessed EN1 expression in four glioma cell lines, and we found that U-118 MG, U251, and A172 had higher EN1 expression than did the U-87 MG cell line (Figure 1E). We next used EN1-shRNA to knockdown (KD) the expression of EN1 in the three glioblastoma cell lines with relatively high EN1 expression, which led to a clear inhibition of proliferation (Figure 2A–C and Figure S2A,B). These findings were later supported by our observation of inhibited proliferation of EN1 knockout lines by CRISPR/Cas9 technology (Table S1 and Figure S3). We also observed that deficiency of EN1 reduced the colony formation abilities of U251 cells (Figure 2D). Conversely, we found that overexpression of EN1 did not increase cellular proliferation, but enhanced the clonogenicity of U-87 MG glioma cells (Figure 2H).

We performed wound-healing assays to evaluate the effect of EN1 on cell migration and discovered that EN1 KD significantly repressed glioblastoma cell migration (Figure 2E,F), whereas increased migration was observed for glioblastoma cells with EN1 overexpression (Figure S2C).

Finally, to study the roles of EN1 in tumor formation in vivo, we transplanted EN1 KD and control U251 cells intracranially into nude mice and found that reducing the EN1 level significantly prolonged the survival of tumor-bearing mice (Figure 2G). Collectively, these results indicate that EN1 functions directly in glioblastoma cell growth in vitro and in vivo.

### 2.3. EN1 Knockdown Elevated Intracellular ROS Levels and Susceptibility to γ-ray Irradiation

**Figure 3:**
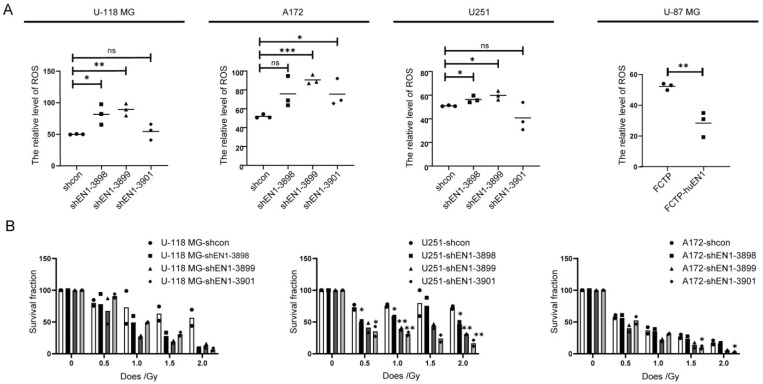
EN1 knockdown increased intracellular ROS levels and elevated susceptibility to γ-ray irradiation. (**A**) EN1 knockdown elevated the intracellular ROS levels of glioma cells, assessed with H2DCFDA assays (● for shcon, ■ for shEN1-3898, ▲ for shEN1-3899, ◆ for shEN1-3901). (**B**) Colony forming assays demonstrating that EN1 knockdown elevated the susceptibility of glioma cells to γ-ray irradiation, assessed with plating efficiency (PE), where PE is the fraction of colonies counted divided by cells plated without radiation. Data are presented as the mean from three independent experiments. * *p* < 0.05, ** *p* < 0.01, *** *p* < 0.001 according to Student’s *t*-test.

A basal level of reactive oxygen species (ROS) is necessary for cellular proliferation and differentiation [29]; to determine the cellular ROS change after EN1 knockdown, fluorescence sensitive probe H2DCF-DA was used for ROS characterization by flow cytometry measurement. The analyses demonstrated that EN1 knockdown caused ROS increase in glioma cells compared with scramble controls (Figure 3A). In contrast, overexpression of EN1 reduced ROS generation (27.15% vs. 51.6% in the control group, *p* < 0.05), which may allow for cell survival during various stressors. These data demonstrated that EN1 is an important regulator of glioma cell ROS generation.

Radiation therapy usually follows surgery in treatment of glioma, especially for high-grade gliomas [30,31]. Ionizing radiation produces ROS, which induce metabolic oxidative stress and prolonged cell injury [32], and the increased ROS level also inhibits the survival and self-renewal of GSCs [33]. Since EN1 knockdown caused ROS increase, we assessed the impact of modulating EN1 on responsivity to radiotherapy using colony formation assays. U-118 MG, U251, and A172 cells were treated with graded doses of radiation (0, 0.5, 1, 1.5, and 2 Gy). Colony-forming efficiency was assessed 2 weeks later, and the surviving fraction was calculated. The colony number of EN1 KD cell lines after radiotherapy was significantly lower than the scramble control cell lines (Figure 3B), suggesting that EN1 might modulate the cellular radiation sensitivity of glioma cells.

### 2.4. EN1 Increases Hedgehog Signaling Activity in Glioma Cells

**Figure 4:**
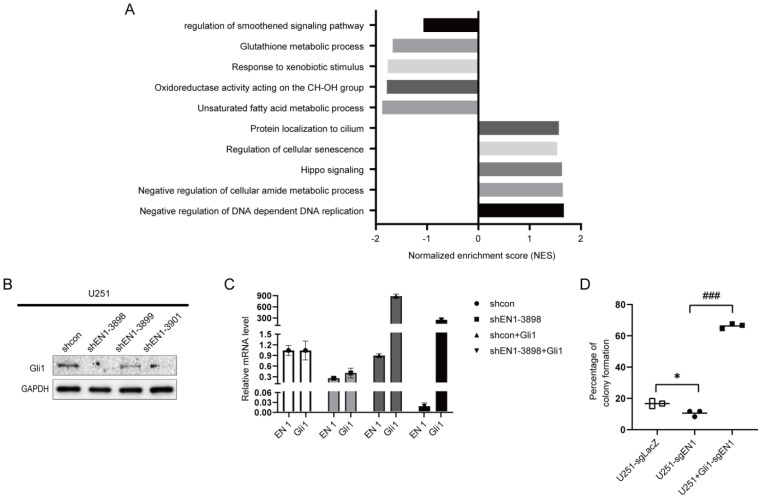
EN1 increases the activity of proliferation related Hedgehog signaling pathways. (**A**) Overrepresented Gene Ontology (GO) terms from RNA−seq analysis of upregulated gene sets and downregulated gene sets in EN1 knockdown group compared to control cells. (**B**) Immunoblotting assays showing that EN1 knockdown significantly reduces the Gli1 level in glioma cells. (**C**) The EN1 and Gli1 transcript levels in U251 cells transduced with scramble control, shEN1, shcon + Gli1, or shEN1 + Gli1. (**D**) Overexpression of Gli1 rescues the restrained colony-forming abilities caused by the knockout of EN1 (□ for U251-sgLacZ, ● for U251-sgEN1, ■ for U251+Gli1-sgEN1). Data are presented as the mean from three independent experiments. * *p* < 0.05, ^###^ *p* < 0.001 according to Student’s *t*-test.

To investigate the potential mechanisms of EN1 in the glioma cell line, we used RNA sequencing to assess glioma cells after knocking down EN1. RNA-seq analysis of these EN1 KD cell lines revealed that EN1 disruption induces differential expression of genes in multiple signaling pathways, including the smoothened signaling pathway (Hedgehog signaling pathway), glutathione metabolic process, protein localization to cilium, regulation of cellular senescence, and DNA replication (Figure 4A).

As the Hedgehog signaling pathway is a major regulator of many fundamental processes including stem-cell maintenance and oncogenesis [34], we next examined the expression of given glioma-associated oncogene homolog 1 (Gli1), which is known to function as a nuclear mediator of the Hedgehog pathway [35]. Gli1 expression was found to be significantly decreased in the EN1 KD cell lines as compared to scramble control cells (Figure 4B and Figure S4). We next carried out functional recovery experiments to determine whether EN1 exerts its functions in glioma cells via the Hedgehog pathway. We evaluated the colony-forming capacity of U251 and U251 overexpressing Gli1 (U251-OE-Gli1) with EN1 knockout mediated by CRISPR/Cas9. Briefly, we found that the restrained colony-forming abilities caused by the knockout of EN1 was reversed in the Gli1-OE cell background (Figure 4C,D). These data suggested that EN1 promotes glioma proliferation by regulating the Hedgehog pathway.

### 2.5. EN1 Knockdown Upregulates the Expression of TULP3

**Figure 5:**
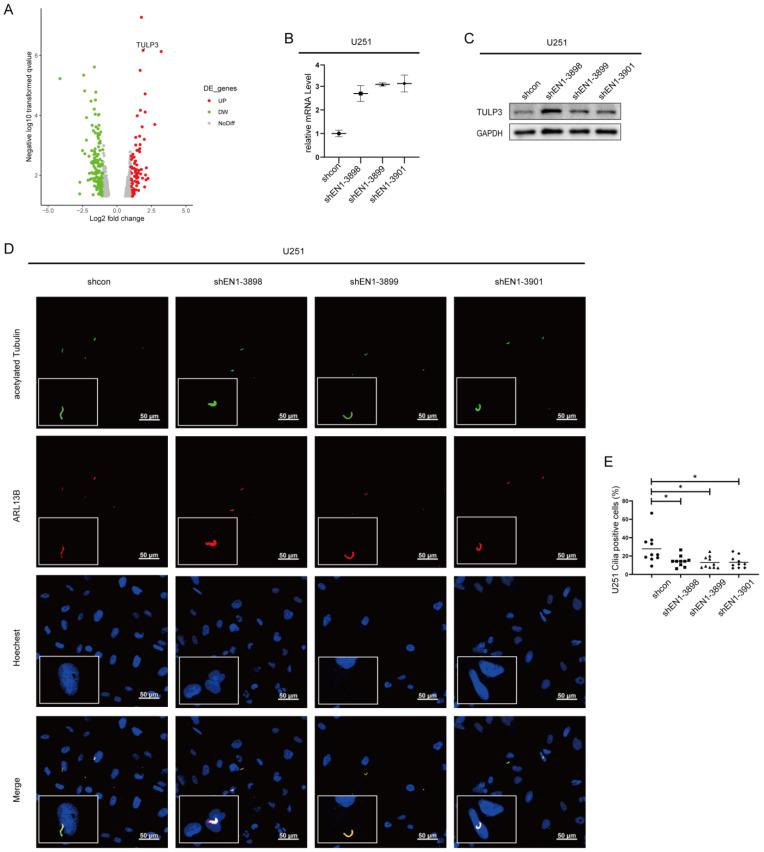
Silencing EN1 upregulates the expression of TULP3. (**A**) Volcano plot of differentially expressed genes (DEGs) in EN1 knockdown glioma cells (vs. scramble control cells). qPCR (**B**) and immunoblotting (**C**) confirmed that EN1 knockdown glioma cells have elevated TULP3. (**D**) Staining of glioma cells with antibodies against ARL13B and acetylated tubulin. (**E**) EN1 knockdown significantly decreased the number of primary cilia. Scale bar: 50 µm. Data are presented as the mean from three independent experiments (● for shcon, ■ for shEN1-3898, ▲ for shEN1-3899, ◆ for shEN1-3901). * *p* < 0.05 according to Student’s *t*-test.

RNA-seq results also revealed that alteration of TULP3 was common to all examined EN1 KD glioma cell lines (Figure 5A and Figure S5A,B). The TULP3 gene product is an adaptor protein that traffics membrane proteins to primary cilia [36], which is the central organelle for the transduction of the Hedgehog signaling pathway [37]. A previous study showed that TULP3 is necessary for the trafficking of Gpr161 to cilia, while Gpr161 functions as a negative regulator of the Sonic hedgehog (SHH) pathway [38]. Furthermore, in mouse embryo studies, TULP3 was similarly described as a negative regulator of Hedgehog signaling [39]. Our qPCR and immunoblotting verified that TULP3 level was significantly increased in EN1 KD cell lines (Figure 5B,C). In addition, we also detected a significant decrease in the number of primary cilia in EN1 KD cells (Figure 5D,E and Figure S5C).

## 3. Discussion

Through cross-species transcriptome studies, we found that EN1 is highly expressed in gliomas, that high expression of EN1 in gliomas affects cell proliferation, colony formation, and resistance to radiotherapy, as well as promoting tumor growth in vivo, and that EN1 may function as an oncogene in gliomas. Therefore, EN1 expression alone can be a strong predictor of patient prognosis; even in patients with glioblastoma, where survival time is often very short, patients with high EN1 expression have significantly worse survival outcomes (Figure 1D).

EN1 has been reported as a key regulator that contributes to the progression of a variety of human cancers, including breast cancer [11,12] and salivary gland adenoid cystic carcinoma [40]. Previous studies found that EN1 could negatively regulate Wnt/β-catenin transcriptional activity in human colon cancer cells [41]. In addition, cytoplasmic or secreted EN1 has been previously reported as a tumor progression marker, while most of its function in tumor development is exercised by repressing or activating the expression of genes in the nucleus. Here, through RNA-seq analysis and further validation, we found that part of the function of EN1 in glioma cells was achieved through regulating SHH pathway activity.

The SHH pathway is also activated in multiple types of tumors, where it affects cancer cell proliferation, growth, and survival [42,43]. Both EN1 and Hedgehog signaling play crucial roles in developmental processes. EN1 is essential in the early establishment and maintenance of the midbrain/hindbrain [44]; interestingly, SHH signaling together with Wnt signaling also plays essential roles in the patterning and development of the midbrain/hindbrain [7]. In *Drosophila*, interactions between Engrailed (en) and Hedgehog signaling pathways revealed that the Hedgehog pathway is transcriptionally activated by the Engrailed protein [45]. Our current study adds further layers to understanding of the regulation of SHH by EN1, through modulating the number of primary cilia and protein trafficking in primary cilia by the TULP3 protein.

In conclusion, our study indicates that EN1 is frequently elevated in glioma tissues and associated with poor prognosis, and it may, therefore, be a promising diagnostic and prognostic marker for glioma patients. Mechanistically, we show that EN1 promotes glioma cell proliferation and migration, doing so at least partially through regulating Hedgehog pathway activity. Taken together, these findings suggest that EN1 is an oncogene in glioma that can be a promising target for the development of innovative therapies against glioma, a deadly disease largely lacking effective treatments.

## 4. Materials and Methods

### 4.1. Cell Lines and Culture

The glioblastoma cell lines U-118 MG, U251, A172, U-87 MG, and T98G were purchased from the Procell Life Science & Technology Co., Ltd. (Wuhan, China). The HEK 293T cell lines were provided by Dr. Ting Han of NIBS (Beijing, China). All cell lines were maintained in Dulbecco’s modified Eagle’s medium (DMEM) (Gibco, Waltham, MA, USA) and were supplemented with 10% fetal bovine serum (FBS) (Gibco, USA) and 1% penicillin/streptomycin antibiotic mixture (Gibco, Waltham, MA, USA). All cells were maintained at 37 °C with 5% CO_2_.

The glioma stem cells were obtained from isolated stem cell cultures of mouse spontaneous glioma tumor samples. The genotypes of the spontaneously tumor-bearing mice were hGFAP-Cre; p53fl/fl; ptenfl/+; nf1fl/+ [24]. The tumor samples were cut into small pieces, digested and blown, and cultured in vitro using glioma stem-cell medium. The cell line obtained after adherent growth was the glioma stem-cell line.

### 4.2. Plasmids and Lentiviral Transduction

The PLKO.1 vector was used to clone the shRNAs targeting EN1 to obtain EN1 knockdown cell lines, and the EN1 gene-deficiency monoclonal cell line was obtained by CRISPR/Cas9-mediated genome editing. For overexpression of EN1 or Gli1 in glioma cells, the full-length EN1 or Gli1 cDNA was amplified and then inserted into the FCTP vector (Table S2 and Figure S6). EN1 loss- and gain-of-function stable cell lines were generated via retroviral infection. Briefly, the HEK 293T cells were transfected with the target plasmids, pSPAX2 and pMD2G, at a ratio of 5:3:2 with Lipofectamine™ 3000 Transfection Reagent (Thermo Scientific, Waltham, MA, USA); virus particles were collected 48 h after transfection. The U-118 MG, U251, U-87 MG, A172, and T98G cells were infected with recombinant lentivirus transducing units using 1 μg/mL polybrene (Sigma-Aldrich, Burlington, MA, USA), and then 1 or 2 μg/mL puromycin (Thermo Scientific, Waltham, MA, USA) was used to screen positive cells for 3 days to acquire stable cell lines.

### 4.3. Quantitative PCR (qPCR)

Total RNA from shRNA-treated and EN1-overexpression cells was isolated from cells using a HP Total RNA Kit (Omega, Boca Raton, FL, USA). cDNAs were synthesized using KOD SYBR qPCR Mix (TOYOBO, Osaka, Japan) according to the manufacturer’s instructions. The 2^−ΔΔCq^ method was used to calculate the expression level; GAPDH was used as a reference gene for quantification assays.

### 4.4. Immunoblotting

Cells were lysed with RIPA buffer, and protein concentrations were determined using the BCA Protein Assay Reagent (Thermo Scientific, Waltham, MA, USA). Equal amounts of protein were subjected to 10% SDS-PAGE before being transferred to a nitrocellulose membrane. The membrane was incubated (overnight, 4 °C) with the following primary antibodies: (EN1-2377, NIBS, Beijing, China), (EN1-2378, NIBS, Beijing, China), (EN1 Polyclonal Antibody, Invitrogen, Waltham, MA, USA), (EN1-4D9, University of California, USA), (EN1-4G11, Columbia University, USA), (EN1, Abcam, Cambridge, MA, USA), (EN1, Sigma-Aldrich, USA), (EN1, Atlas Antibodies, Stockholm, Sweden), (EN1, Cusabio, Wuhan, China) (only EN1-2377 recognizes the correct size of EN1 band), (GAPDH, Origene, USA), and (TULP3, Abcam, Cambridge, MA, USA), (Gli1, Cell Signaling Technology, Danvers, MA, USA), before washing three times with TBST. The membrane was then incubated (1 h) with horseradish peroxidase-conjugated secondary goat anti-rabbit or anti-mouse antibodies (Cell Signaling Technology, Danvers, MA, USA), before being washed again with TBST (Table S3). Signals were detected using an ECL solution (Thermo Fisher, Waltham, MA, USA).

### 4.5. Cell Proliferation Assays

The effect of EN1 on cell proliferation was measured using a CellTiter-Glo assay kit (Promega, Madison, WI, USA). The glioblastoma cells were seeded at a density of 2500 cells in 96-well plates and incubated. CellTiter-Glo^®^ reagents were mixed and added to the wells after 24/72/120 h. After 2 min of mixing and 10 min of incubation at room temperature, luminescence was recorded on an EnSpire^®^ Multimode Plate Reader (PerkinElmer, Waltham, MA, USA). The luminescence was normalized to the value assessed on day 0, and it is presented as the mean to show the relative proliferation rates of cells after different incubation times. Each experiment was performed in triplicate.

For the EdU incorporation assay, the 5 × 10^4^ scramble control and EN1-KD cells were cultured in six-well plates for 16 h at 37 °C and then exposed to 20 µM 5-ethynyl-2′-deoxyuridine (EdU, Beyotime, Beijing, China) for an additional 3 h. The cells were fixed with 4% formaldehyde for 20 min and permeabilized with 0.5% Triton X-100 at room temperature for 10 min. After washing three times with PBS, Click Reaction Buffer (100 µL) was added to each well and incubated for 30 min, while being protected from light. The cells were subsequently stained with Hoechst 33342 for 10 min, washed three times with PBS, and visualized under a Nikon fluorescent microscope (Nikon, Tokyo, Japan). The EdU-positive cells (green cells) were counted using NIS-Elements BR 3.0 software (Nikon, Tokyo, Japan). The EdU incorporation rate was expressed as the ratio of the number of EdU-positive cells (green cells) to the total number of Hoechst 33342-positive cells (blue cells).

### 4.6. Wound-Healing Assay

The effect of gene silencing on cell proliferation was measured using a wound-healing assay [46]. Glioblastoma cells were seeded in six-well plates and incubated to reach 90% confluence. Monolayers were scratched with a sterile 200 µL pipette tip to create a wound, and cells were then washed twice with PBS to remove floating cells. Subsequently, cells were cultured in 2% serum DMEM medium for an additional 48 h. Images were captured using a microscope at 0 and 24/48 h, and they were analyzed using the wound-healing macro of ImageJ. The rate of wound closure was calculated as a function of the area of the gap.

### 4.7. Colony Formation Assays

To evaluate the effect of EN1 in the response to radiotherapy, glioblastoma cells were treated using a γ-ray generator Gammacell 1000 (MDS Nordion, Ottawa, ON, Canada) with doses ranging 0–2 Gy. After different doses of γ-ray irradiation, the glioblastoma cells were suspended and plated in 6 cm plates at 1000 cells/plate for colony formation. Two weeks later, the colonies were fixed in paraformaldehyde (PFA) 4%, then stained with 0.5% crystal violet for 30 min, and photographed. The colonies with more than 50 cells were identified and enumerated using an inverted microscope. Survival was calculated as the average number of colonies counted divided by the number of cells plated multiplicated by plating efficiency (PE), where plating efficiency (PE) is defined as the number of colonies observed/the number of cells plated without radiation [47].

### 4.8. ROS Level

The scramble control and EN1-KD cell intracellular ROS levels were measured and analyzed by the DCFDA/H2DCFDA assay. Cells were harvested and trypsinized as a single cell, washed with DPBS to remove traces of the original medium, resuspended in 10 μM H2DCFDA (Thermo Fisher Scientific, Waltham, MA, USA) in DMEM medium without serum, and stained for 25 min in the dark at 37 °C. After staining, cells were washed twice with PBS and then resuspended in PBS. H2DCFDA fluoresce signaling was detected by flow cytometer (BD Biosciences, San Jose, CA, USA) at the 488 nm channel, and a total of 10,000 cells were analyzed per sample. Data analysis was performed with FlowJo 7.6 software. Relative fluorescence was calculated by normalization to the control group.

### 4.9. Immunofluorescence

For immunostaining, the glioma cells were seeded in six-well plates with coverslips (10^5^ cell/well) overnight and fixed in 4% paraformaldehyde. After permeabilizing with 0.25% Triton X-100 for 10 min, cells were blocked in 10% goat serum and incubated with primary antibodies (acetylated tubulin (Lys40), Mouse mAb; ARL13B, Rabbit pAb) (overnight, 4 °C) and washed (30 min) with 0.1% Triton X-100 in PBS. The cells were then incubated for 2 h at room temperature with a secondary Alexa Fluor™ 594 goat anti-mouse IgG (H + L) antibody or 488 goat anti-ribbit IgG (H + L) antibody (Invitrogen, Waltham, MA, USA) and washed again (30 min) with 0.1% Triton X-100 in PBS. Slides were mounted using Prolong Gold antifade with DAPI. Images were acquired using a Nikon fluorescent microscope (NIKON, Tokyo, Japan). The cilia positive cells were expressed as the ratio of the number of cilia-positive cells (green + red cells) to the total number of Hoechst 33342-positive cells (blue cells), counting at least 100 cells

Due to antibody limitations, we could not perform the experiments of detect EN1 expression by IHC, Meanwhile, we checked the single-cell RNA-seq data of glioblastoma [48] and confirmed that EN1 and EN2 was mostly expressed by the tumor cells, and that the EN1 level was higher than EN2 (Figure S7).

### 4.10. RNA-Seq

Total RNA was isolated from cell samples with Trizol reagent according to the manufacturer’s protocol and sequenced by GENEWIZ (China). Reads were mapped to GRCh37.p13 genome and annotated with STAR, using the ENCODES recommended arguments. Gene-level read counts generated by STAR were used for differential expression analysis with the DESeq2 package. Log_2_ fold change and adjusted *p*-values of genes generated by DESeq2 were used for visualization using R.

### 4.11. Bioinformatics Analysis

TCGA glioma dataset was retrieved from Broad GDAC Firehose (https://gdac.broadinstitute.org/, data version 28 January 2016), and the Henry Ford Hospital dataset was retrieved from the Gene Expression Omnibus (GEO) with accession number GSE4290 following by quantile normalization and log_2_ transformation with R software. The dataset with 525 glioma cancer samples and 148 normal glioma samples was retrieved from GEO (GSE16011), which were used to detect the expression of EN1 and EN2 in glioma samples. Next, we verified the expression of EN1 in glioma tissues in CGGA (CGGA, Chinese Glioma Genome Atlas; http://cgga.org.cn, accessed on 8 December 2021, DataSet ID: mRNAseq_325) (325 samples) and the Henry Ford Hospital datasets. The expression of EN1 was evaluated with means and standard errors of the mean (SEM) with Graphpad Prism Software 8.0 (San Diego, CA, USA). The power of EN1 to differentiate glioma cancer from normal tissues was evaluated according to ROC curves. We performed correlation analysis to screen co-expression genes of EN1 in glioma patients, with screening criteria as follows: *p* < 0.05 and |Pearson correlation coefficient| ≥ 0.3. To determine how EN1 affected the prognosis of glioma patients, we performed Kyoto Encyclopedia of Genes and Genomes (KEGG) enrichment analysis of EN1 co-expressed genes.

### 4.12. Animal Studies

All animal experiments were conducted following the National Guidelines for Housing and Care of Laboratory Animals in China and performed under the approved Institutional Animal Care and Use Committee protocols.

For mouse tumor models, U251-shcon, U251-shEN1-3898, or U251-shEN1-3899 cells were administered intracranially into the 6–8 week old female BALB/c nude mice (*n* = 5). The cells were suspended in phosphate-buffered saline (Thermo Scientific, Waltham, MA, USA): Matrigel (Corning, NY, USA) = 1:1 culture medium (5 × 10^5^ cells/5 µL) and injected with a glass micropipette at a rate of 1 µL/min with a Hamilton syringe (10 µL) and a stereotaxic microinjector (RWD, Shenzhen, China). The animals were then monitored daily, and mice were killed when they had a weight decrease >30%. The survival of each mouse was recorded and overall survival was calculated.

### 4.13. Statistical Analyses

All statistical analyses in this study were conducted using GraphPad Prism 8.0 software (GraphPad, USA). For experimental work, all assays were performed in triplicate, and the measurement data are presented as the mean ± standard deviation (SD). Statistical comparisons between two groups were based on Student’s *t*-tests, with multiple *t*-test used for multigroup comparisons. A *p*-value < 0.05 was considered statistically significant.

## Data Availability

All data are contained within this manuscript.

## Supplementary Materials

The following supporting information can be downloaded at https://www.mdpi.com/article/10.3390/ijms23031123/s1.
