# Supplementary material for: EN1 Regulates Cell Growth and Proliferation in Human Glioma Cells via Hedgehog Signaling
Source: Int J Mol Sci. 2022 Jan 20;23(3):1123. doi: 10.3390/ijms23031123 (PMC8834903; doi:10.3390/ijms23031123)
Supplement: Supplementary file 1 [file ijms-23-01123-s001.zip › ijms-1509734-supplementary.pdf]

IJMS

Supplemental Information

EN1 regulates cell growth and proliferation in human glioma cells via Hedgehog signaling

Jinchun Chang, Chenjia Guo, Jianyu Li, Zhangqian Liang, Yankai Wang, Anliang Yu, Runze Liu, Yuting Guo, Jian Chen,

Song Huang

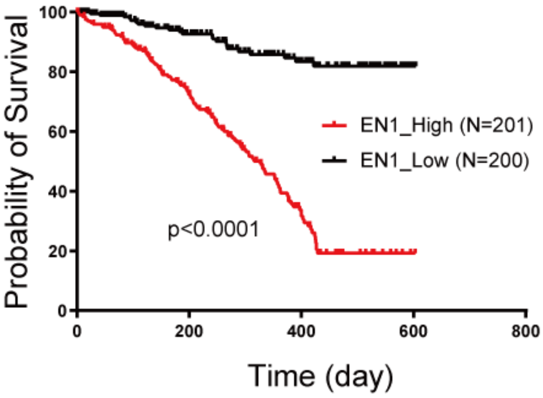

Figure S1 Prognosis result of EN1 expression in all the GBMLGG data set from TCGA.

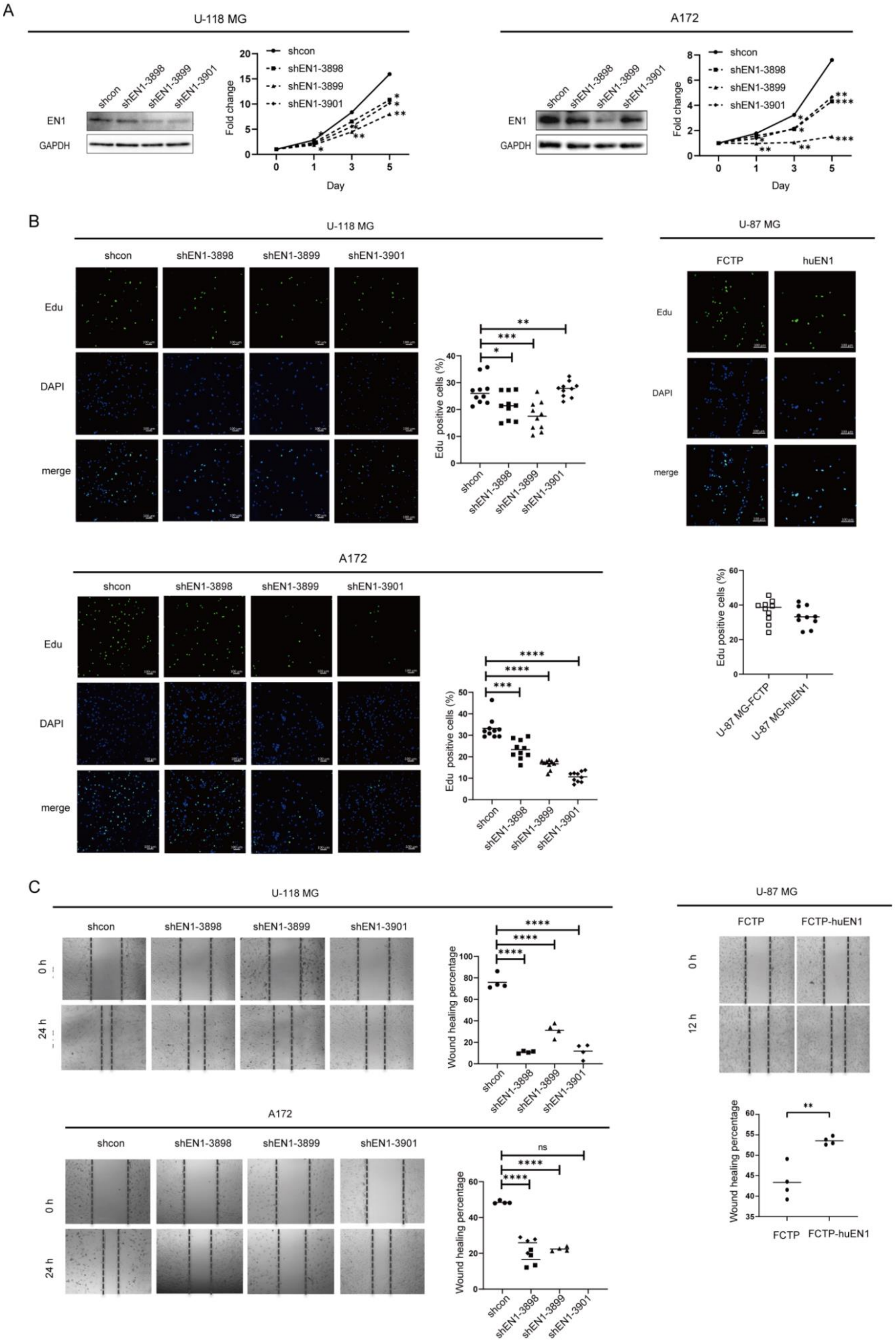

Figure S2 EN1 knockdown restrains the proliferation and migration of glioma cells. **A** Assessment of knockdown efficiency for EN1 in U-118 MG and A172 cells with immunoblotting and cell proliferation was assessed using CellTiter-Glo assays. 5-Ethynyl-2'-deoxyuridine (Edu) assays (**B**) and wound healing assay (**C**) was used in U-118 MG,

A172 and U-87 MG cells to assessment the effect of EN1 on cell proliferation and migration. Scale bar: 50 μm. Data are presented as the mean from three independent experiments. * $p < 0.05$, ** $p < 0.01$, *** $p < 0.001$ by Student's t-test.

Table S1 Variation of the percentage of RFP/muGFP (%) cells after 2, 9, 16 days of CRISPR-cas9 system action.

	Cell line	Day 2	Day 9	Day 16
U-118 MG	RFP-LacZ(NC)	#	#	#
	muGFP-LacZ(NC)	0	0	0
	RFP+PSMD1(PC)	101.446	103.713	129.474
	RFP+EN1 1-1	84.153	85.472	89.461
	RFP+EN1 1-2	103.282	97.217	122.506
	RFP+EN1 1-3	103.140	101.331	118.124
	RFP+EN1 1-4	79.160	98.929	87.811
U251	RFP-LacZ(NC)	#	#	#
	muGFP-LacZ(NC)	0	0	0
	RFP+PSMD1(PC)	111.852	138.742	223.332
	RFP+EN1 1-1	57.135	106.947	184.520
	RFP+EN1 1-2	65.924	120.655	191.119
	RFP+EN1 1-3	82.627	118.635	176.083
	RFP+EN1 1-4	49.907	94.970	198.915
T98G	RFP-LacZ(NC)	#	#	#
	muGFP-LacZ(NC)	0	0	0
	RFP+PSMD1(PC)	32.295	55.373	98.765
	RFP+EN1 1-1	31.050	52.337	74.328
	RFP+EN1 1-2	33.246	54.336	90.794
	RFP+EN1 1-3	54.238	67.720	94.470
	RFP+EN1 1-4	31.199	53.752	72.446

A

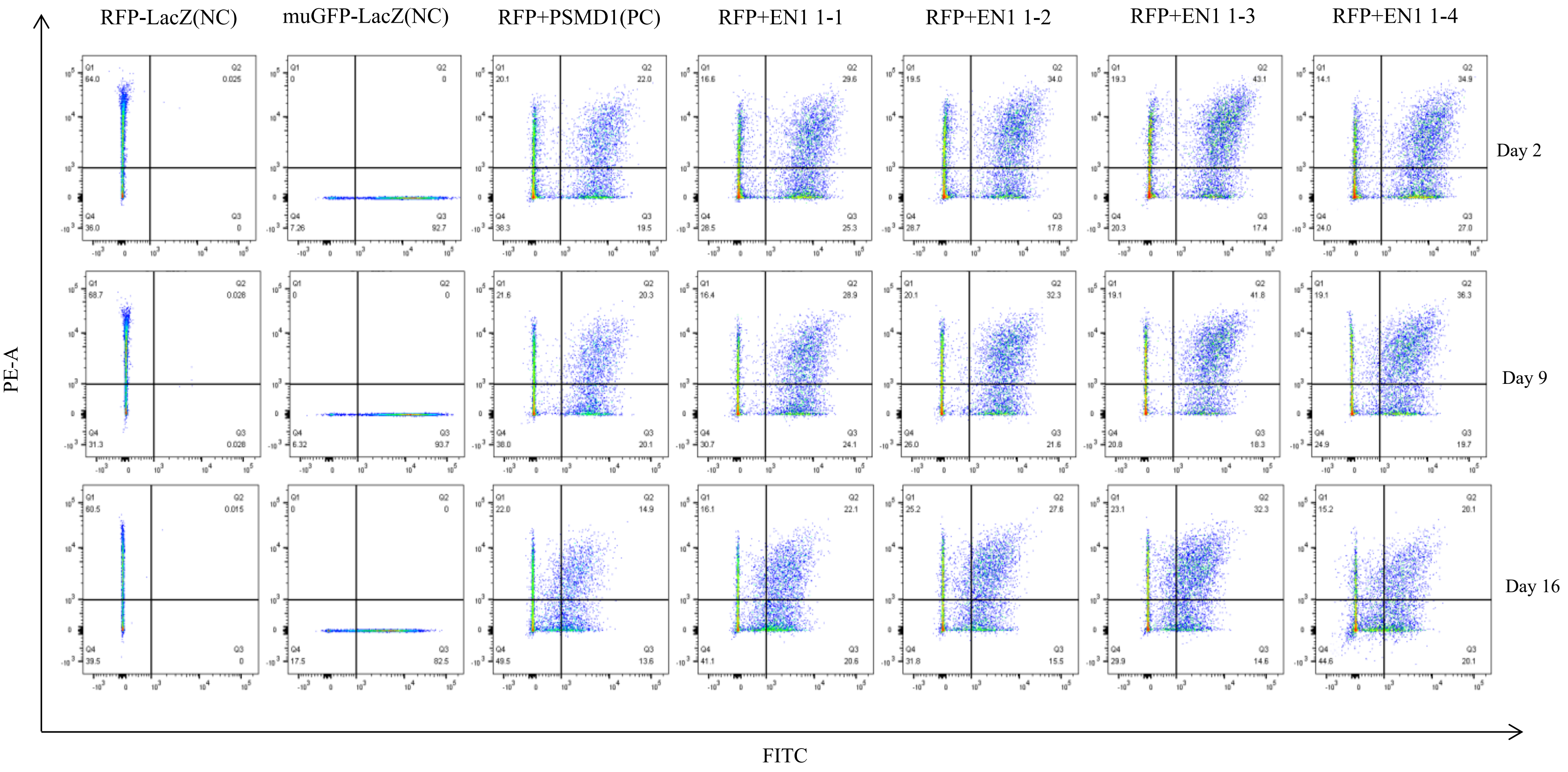

RFP-LacZ(NC)
muGFP-LacZ(NC)
RFP+PSMD1(PC)
RFP+EN1 1-1
RFP+EN1 1-2
RFP+EN1 1-3
RFP+EN1 1-4
Day 2
Day 9
Day 16
PE-A
FITC

B

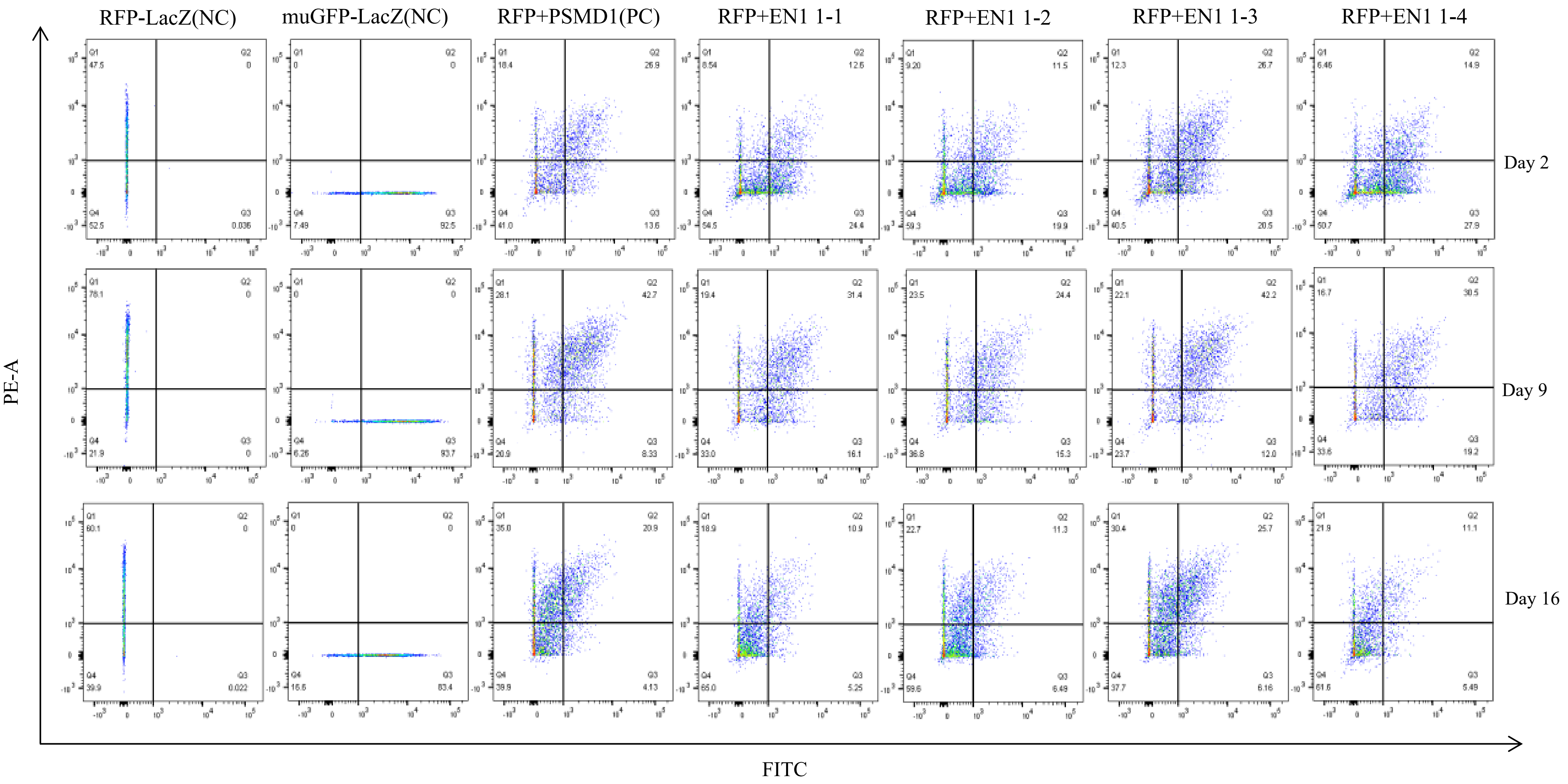
RFP-LacZ(NC)
muGFP-LacZ(NC)
RFP+PSMD1(PC)
RFP+EN1 1-1
RFP+EN1 1-2
RFP+EN1 1-3
RFP+EN1 1-4
Day 2
Day 9
Day 16
PE-A
FITC

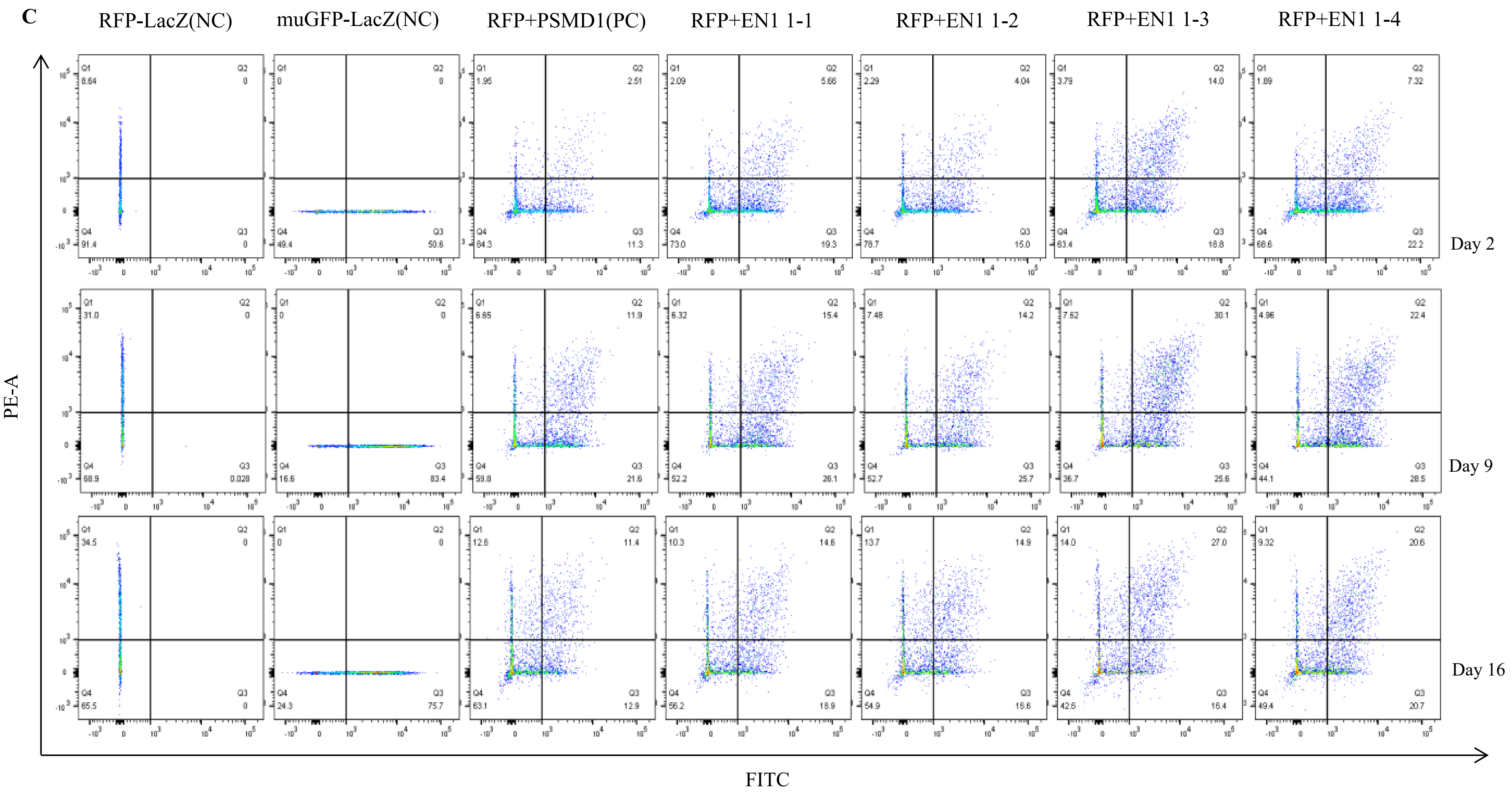
C
RFP-LacZ(NC)
muGFP-LacZ(NC)
RFP+PSMD1(PC)
RFP+EN1 1-1
RFP+EN1 1-2
RFP+EN1 1-3
RFP+EN1 1-4
PE-A
Day 2
Day 9
Day 16
FITC

Figure S3 Loss of function of EN1 leads to a decrease in cell proliferation or an increase in cell death. CRISPR-Cas9/gRNA systems have been designed to detecting the impact of deficiencies of certain genes on cell proliferation. Cas9+ glioma cells infected with different sgRNA with RFP or muGFP labeling (sgLacZ as a negative control, sgPSMD1 as a positive control, four sgRNAs targeting EN1 as experimental groups), flow cytometry analysis of RFP/muGFP percentage after EN1 knock out in the U-118 MG, U251, and T98G glioma cell lines at 2, 9, 16 days respectively. **A** for the U118 cell line. **B** for the U251cell line. **C** for the T98G cell line.

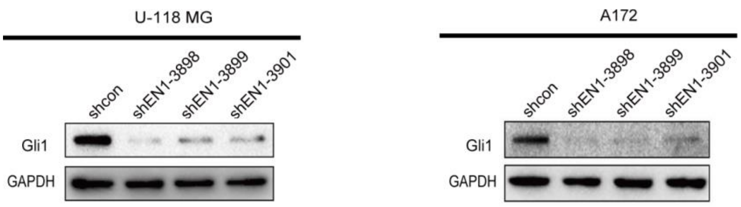

Figure S4 Immunoblotting assays showing that EN1 knockdown significantly reduces the Gli1 level in U-118 MG, A172 glioma cells.

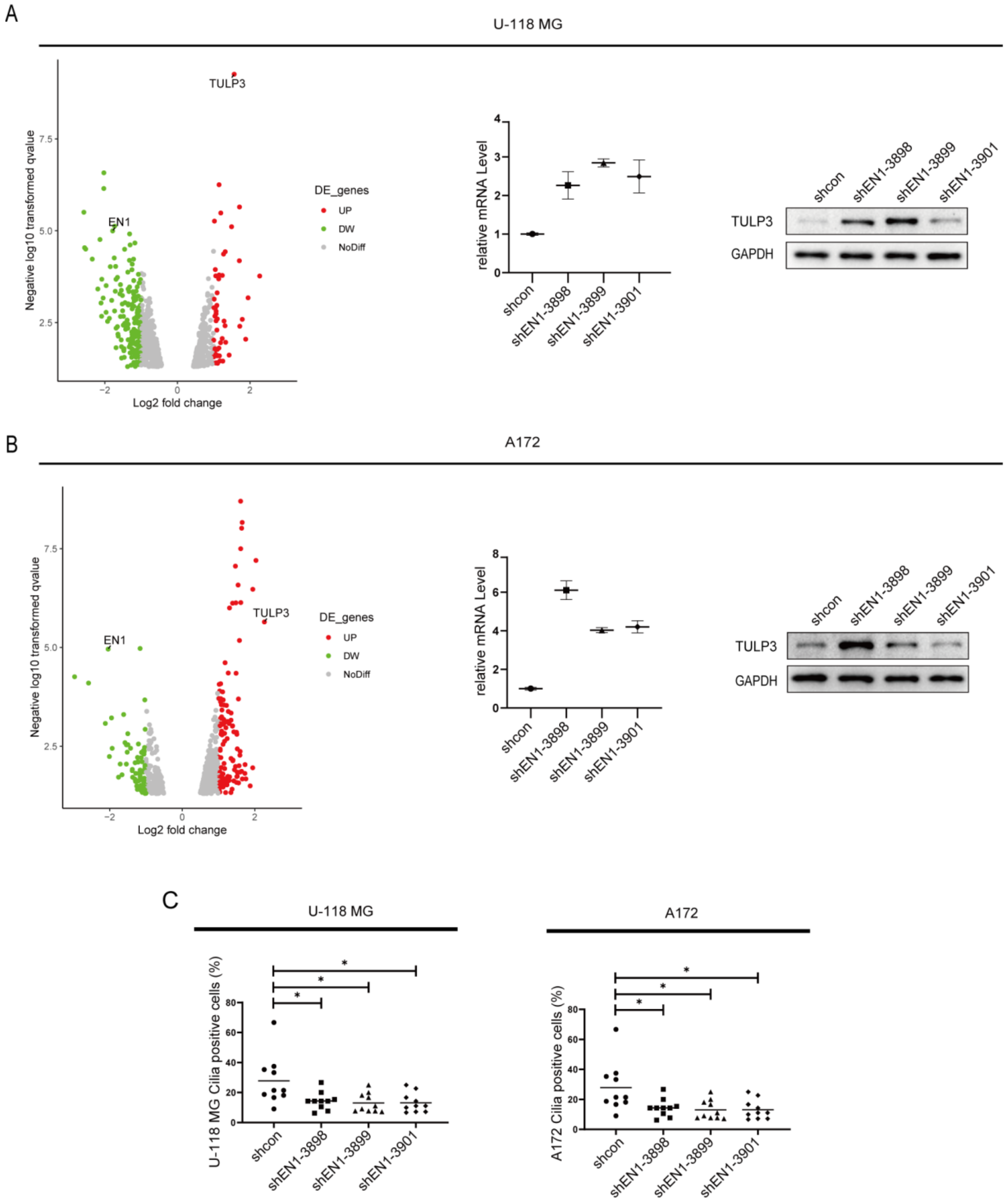

Figure S5 Silencing EN1 upregulates the expression of TULP3. Volcano plot of differentially expressed genes (DEGs) in EN1 knockdown glioma cells (vs. scramble control cells), qPCR and immunoblotting confirmed that EN1 knockdown glioma cells have elevated TULP3. U-118 MG (**A**), A172 (**B**). **C** EN1 knockdown significantly decreased number of primary cilia in U-118 MG, A172 glioma cells. Data are presented as the mean from three independent experiments. * $p < 0.05$, ** $p < 0.01$, *** $p < 0.001$ by Student's t-test.

Table S2 List of primers used in this article.

Gene	Primer	Nucleotide sequence 5′-3′	Purpose
plko-seq	Forward	TTATGTTTTAAAATGGACTATCA	ShRNA Sequencing
shcon	Forward	CCGGCAACAAGATGAAGAGCACCAACTCG AGTTGGTGCTCTTCATCTTGTTGTTTTTG	shRNA
	Reverse	AATTCCAAAAACAACAAGATGAAGAGCAC CAACTCGAGTTGGTGCTCTTCATCTTGTTG	shRNA
ShRNA-EN1-3898	Forward	CCGGCCATTCTCTATTCCCAGTATACTCGAG TATACTGGGAATAGAGAATGGTTTTTG	shRNA
	Reverse	AATTCCAAAAACCATTCTCTATTCCCAGTAT ACTCGAGTATACTGGGAATAGAGAATGG	shRNA
ShRNA-EN1-3899	Forward	CCGGCAACCCGGCTATCCTACTTATCTCGAG ATAAGTAGGATAGCCGGGTTGTTTTTG	shRNA
	Reverse	AATTCCAAAAACAACCCGGCTATCCTACTT ATCTCGAGATAAGTAGGATAGCCGGGTTG	shRNA
ShRNA-EN1-3901	Forward	CCGGGATCAAGAAAGCCACAGGCATCTCGA GATGCCTGTGGCTTTCTTGATCTTTTTG	shRNA
	Reverse	AATTCCAAAAAGATCAAGAAAGCCACAGG CATCTCGAGATGCCTGTGGCTTTCTTGATC	shRNA
sgRNA-huEN1-1	Forward	GGCTTTATATATCTTGTGGAAAGGACGAAA CACCGGCGAACTGTGGCCCACCCGA	sgRNA
	Reverse	GCCTTATTTTAACTTGCTATTTCTAGCTCTAA AACTCGGGTGGGCCACAGTTCGC	sgRNA
sgRNA-huEN1-2	Forward	GGCTTTATATATCTTGTGGAAAGGACGAAA CACCGGCGCGACTCGGCCCTCGGCG	sgRNA
	Reverse	GCCTTATTTTAACTTGCTATTTCTAGCTCTAA AACCGCCGAGGGCCGAGTCGCGC	sgRNA
sgRNA-huEN1-3	Forward	GGCTTTATATATCTTGTGGAAAGGACGAAA CACCGCCTGGCGCCCGGGTGCCCAA	sgRNA
	Reverse	GCCTTATTTTAACTTGCTATTTCTAGCTCTAA AACTTGGGCACCCGGGCGCCAGG	sgRNA
sgRNA-huEN1-4	Forward	GGCTTTATATATCTTGTGGAAAGGACGAAA CACCGGATGTTGTCGATGAAAAAGT	sgRNA
	Reverse	GCCTTATTTTAACTTGCTATTTCTAGCTCTAA AACACTTTTTCATCGACAACATC	sgRNA
HuEN1	Forward	AAGAGATCCAGTGCACCCTC	Sequencing
	Reverse	CCTCCTCCTCCTGTATCGCT	Sequencing
Gli1	Forward	TACTTCGCCCACTGCAGCAT	Sequencing
	Reverse	GACTGAGGATGTGGGATCAT	Sequencing
	Reverse	GTGCGTCTTCAGGTTTTCGAGG	Sequencing
	Reverse	CCCCTGCATTGGGGTTGTATC	Sequencing
TULP3	Forward	CTGCCCCTCAAGGTGTCACA	Sequencing
	Reverse	GATCAATGGAGATAAGGTAGTTGGCTG	Sequencing
HuEN1	Forward	CCGGAATTCCGGGCCACCATGGAGGAAC	Amplification
	Reverse	CGCGGATCCGCG	Amplification

Gene	Primer	Nucleotide sequence 5′-3′	Purpose
		AGCGTAATCTGGAACATCGTA	
Gli1	Forward	CTAGTCTAGAATGTTCAACTCGATGACCCCA CCACCAATC	Amplification
	Reverse	TTGGCGCGCCTTAGGCACTAGAGTTGAGGA ATTCTG	Amplification
TULP3	Forward	CTAGTCTAGACTAGATGGAGGCTTCGCGCTG CCGGC	Amplification
	Reverse	CGCGGATCCGCGTCATTCACACGCCAG	Amplification
HuGAPDH	Forward	AATCCCATCACCATCTTCCA	qPCR
	Reverse	TGGACTCCACGACGTACTCA	qPCR
HuEN1	Forward	ATCGTCCATCCTCCGGTCC	qPCR
	Reverse	GAACTCCGCCTTGAGTCTCT	qPCR
HuEN2	Forward	CCGGCGTGGGTCTACTGTA	qPCR
	Reverse	CCTCTTTGTTCGGGTTCTTCTT	qPCR
PTCH1	Forward	GAAGAAGGTGCTAATGTCCTGAC	qPCR
	Reverse	GTCCCAGACTGTAATTTCGCC	qPCR
SMO	Forward	GAAGTGCCCTTGGTTCGGA	qPCR
	Reverse	GCAGGGTAGCGATTCGAGTT	qPCR
GLI1	Forward	AGCGTGAGCCTGAATCTGTG	qPCR
	Reverse	CAGCATGTACTGGGCTTTGAA	qPCR
GLI2	Forward	CTGCCTCCGAGAAGCAAGAAG	qPCR
	Reverse	GCATGGAATGGTGGCAAGAG	qPCR
TULP3	Forward	TGAGCCATTTATGGTGCAGC	qPCR
	Reverse	GCAGTATCCACGGTGTTTTCAG	qPCR
SHH	Forward	CCAAGGCACATATCCACTGCT	qPCR
	Reverse	GTCTCGATCACGTAGAAGACCT	qPCR

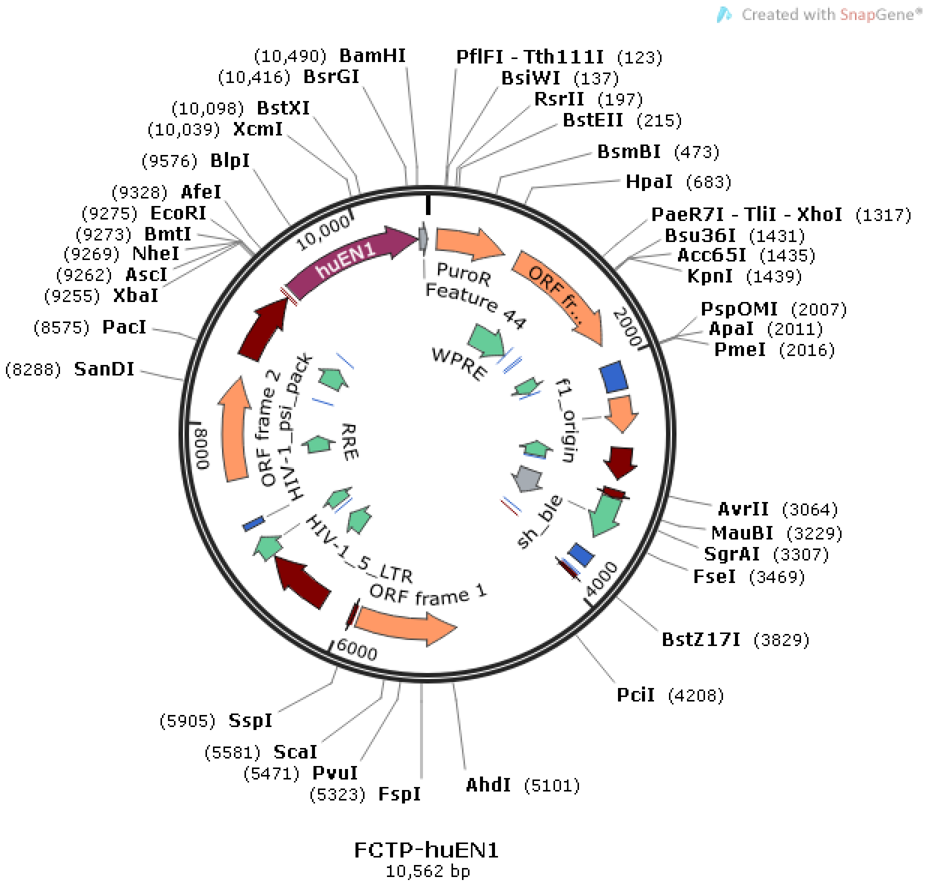

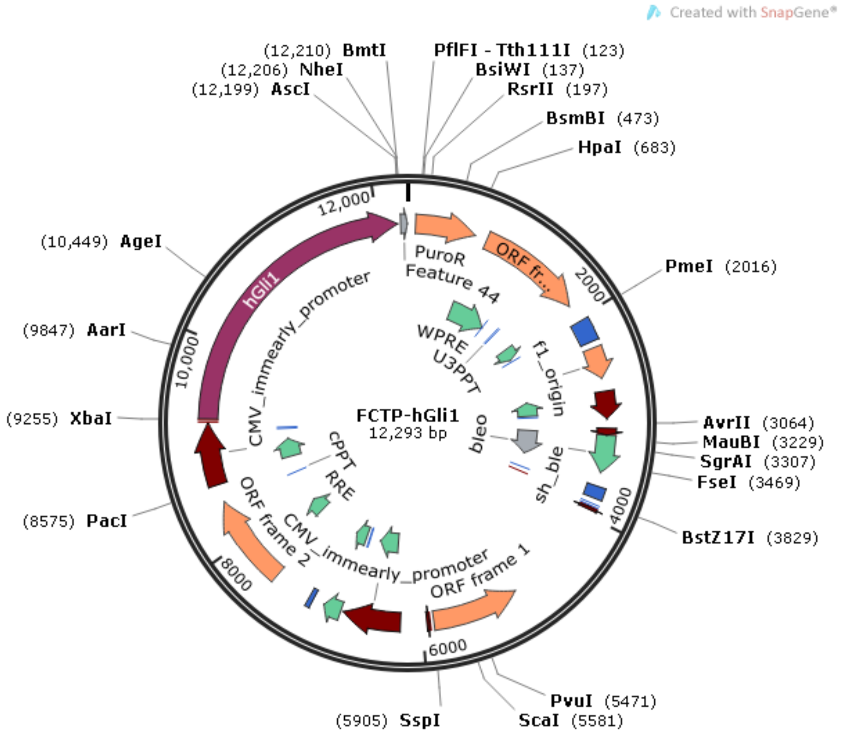

Figure S6 Schematic diagram of plasmid FCTP-huEN1 and FCTP-hGli1 structure.

Table S3 List of antibody used in this article.

Antibody	Company	Catalog number
EN1-2377	NIBS	2377
EN1-2378	NIBS	2378
EN1 Polyclonal Antibody	Invitrogen	# PA5-14149
Engrailed/invected gene products antibody	University of California	4D9
Engrail-1 antibody	Columbia University	4G11
Anti-EN1 / Engrailed 1 antibody	Abcam	ab70993
Anti-EN1 / Engrailed 1 antibody	Abcam	ab83693
Anti-EN1 / Engrailed 1 antibody	Abcam	ab108598
Monoclonal Anti-EN1	Sigma-Aldrich	SAB1403771-100UG
Anti-EN1 Antibody	Atlas Antibodies	HPA073141
Anti-EN1 Polyclonal Antibody	Cusabio	CSB-PA007659LA01CH
GAPDH Mouse Monoclonal Antibody	origene	TA802519
GLI1 (C68H3) Rabbit mAb	Cell Signaling Technology	#3538
Shh (C9C5) Rabbit mAb	Cell Signaling Technology	#2207
TULP3 Mouse Monoclonal Antibody	origene	TA504148
Anti-mouse IgG, HRP-linked Antibody	Cell Signaling Technology	7076S
Anti-rabbit IgG, HRP-linked Antibody	Cell Signaling Technology	7074S
ARL13B	proteintech	17711-1-AP
acetylated Tubulin(Lys40)	proteintech	66200-1-Ig
Goat anti-Mouse IgG (H+L), Alexa Fluor 594	Invitrogen	# A-11005
Goat anti-Rabbit IgG (H+L), Alexa Fluor 488	Invitrogen	# A-11008

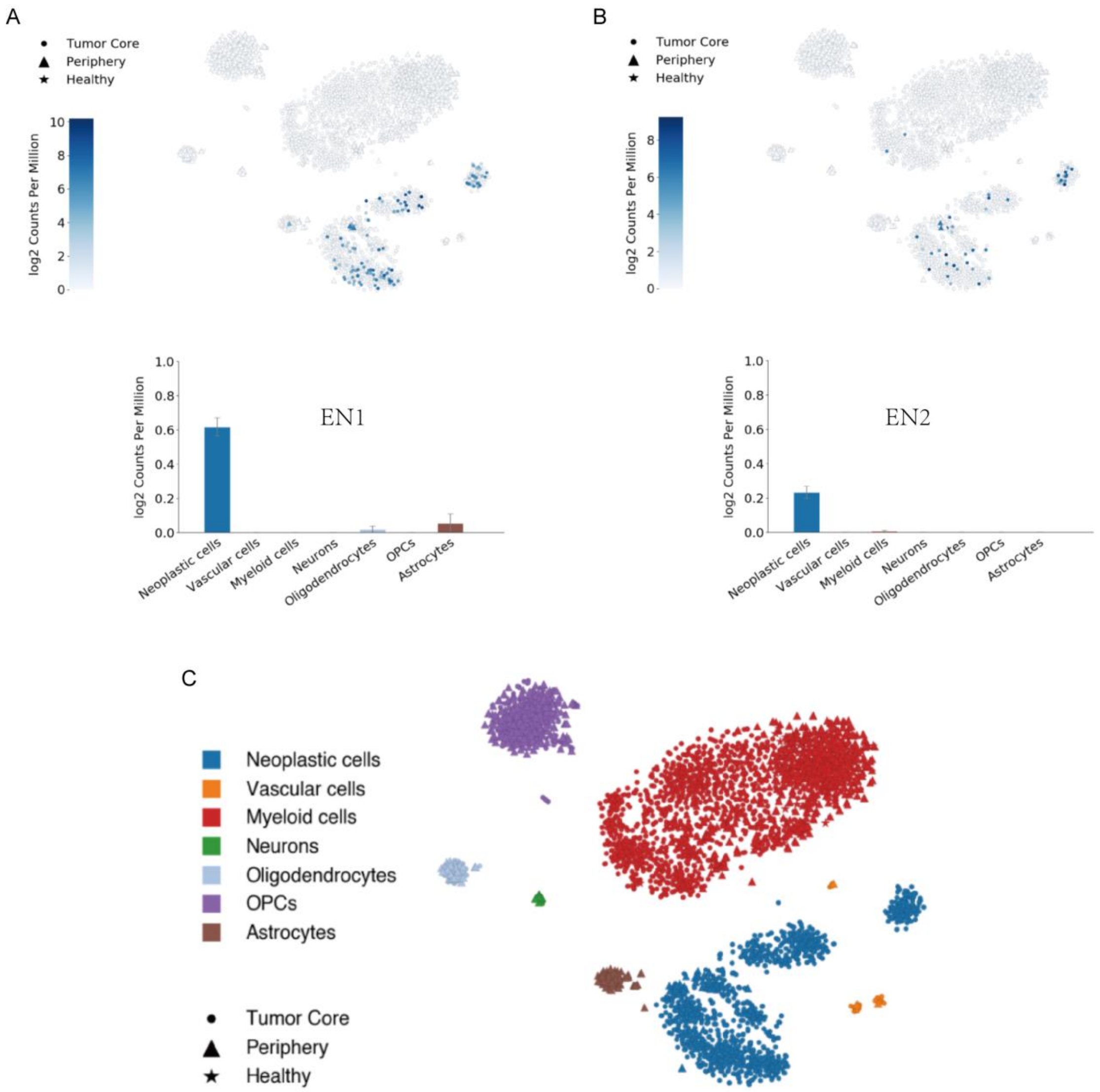

Figure S7 EN1 and EN2 is mainly expressed by GBM tumor cells. A & B Cell type and expression level of EN1 (**A**) and EN2 (**B**) expression in GBM single cell RNAseq. **C** Cell type annotation by single cell RNAseq of the GBM sample used for **A & B.**
